# Individual, household and contextual factors associated with skilled delivery care in Ethiopia: Evidence from Ethiopian demographic and health surveys

**DOI:** 10.1371/journal.pone.0184688

**Published:** 2017-09-14

**Authors:** Markos Mezmur, Kannan Navaneetham, Gobopamang Letamo, Hadgu Bariagaber

**Affiliations:** Department of Population Studies, University of Botswana, Gaborone, Botswana; University of West London, UNITED KINGDOM

## Abstract

Despite evidence that social contexts are key determinants of health, research into factors associated with maternal health service utilization in Ethiopia has often focused on individual and household factors. The downside is that this underestimates the importance of taking contextual factors into account when planning appropriate interventions in promoting safe motherhood in the country. The purpose of this study is to fill this knowledge gap drawing attention to the largely unexplored contextual factors affecting the uptake of skilled attendance at delivery in a nationally representative sample. Data for the study comes from two rounds of the Ethiopian Demographic and Health Surveys (EDHS) conducted in the year 2005 and 2011. Analysis was done using a two-level multivariable multilevel logistic regression model with data from 14, 242 women who had a live birth in the five years preceding the surveys clustered within 540 (in the year 2005) and 624 (in the year 2011) communities. The results of the study point to multiple levels of measured and unmeasured factors affecting the uptake of skilled delivery care in the country. At community level, place of residence, community level of female education and fertility significantly predict the uptake of skilled delivery care. At individual and household level, maternal age, birth order, maternal education, household wealth and access to media predict the uptake of such service. Thus, there is a need to consider community contexts in the design of maternal health programs and employ multi-sectorial approach to addressing barriers at different levels. For example, improving access and availability of skilled delivery care should eventually enhance the uptake of such services at community level in Ethiopia. At individual level, efforts to promote the uptake of such services should constitute targeted interventions paying special attention to the needs of the youth, the multiparous, the less educated and women in the poorest households.

## Introduction

Despite the significant global achievements in promoting maternal survival in the developing region over the past 25 years, maternal health continues to remain a major public health and development challenge for many countries. Worldwide, an estimated 289,000 maternal deaths occurred in 2013 and this represents a decline by 50% from an estimated 523, 000 maternal deaths in 1990 [1]. The highest rates of maternal mortality, to date, are concentrated in Sub-Saharan Africa and South Asia. These two regions of the world account for 85% of the global burden of maternal mortality. Sub-Saharan Africa alone accounts 56% with a maternal mortality ratio (MMR) of 500 per 100, 000 live births [2]. Maternal mortality in Ethiopia is one of the highest in Sub-Saharan Africa. MMR in the year 2011 was estimated 676 per 100,000 live births suggesting virtually no evidence of decline from the level of 673 per 100,000 live births in the year 2005. The figure is much higher compared to the 510 such deaths per 100,000 live births in Sub Saharan Africa for the same year [3].

Several studies have examined the uptake of skilled delivery care in Ethiopia [4–8]. One important inference from existing literature is thus influences of individual and household level factors on the uptake of such services vary across geographic and social settings. However, these studies focused on individual level factors largely ignoring influences contextual factors exert on the uptake of health care services. The downside is that this underestimates the importance of taking contextual factors into account when planning appropriate interventions in promoting safe motherhood in the country. In recent years, studies have also found that contextual factors are key determinants of maternal health service utilization [9–15]. The argument put forward is that persons with similar socio-economic characteristics may have different health seeking behavior depending on whether they live in one community or another and therefore the contextual phenomena that cluster individual health seeking behavior within communities has become a core notion of social epidemiology literature [16, 17].

For example, research in sub-Saharan Africa has shown that poor mothers in communities of high literacy are more likely to use maternal health services than their counterparts in lower educated communities [18]. In a study in Tanzania, poorer households in wealthy regions are found to be better off in maternal health care service utilization outcomes than poorer households in poorer regions [19]. In the context of Ethiopia, little is known about the association of contextual level factors with the uptake of skilled delivery care. The purpose of this study is to fill this knowledge gap drawing attention to the largely unexplored contextual factors affecting the uptake of skilled delivery care in a nationally representative sample using two rounds of Ethiopian Demographic and Health Surveys (DHS). As such the study further contributes in understanding how effects of individual and community level factors on the uptake of skilled delivery care changes over time in the context of Ethiopia.

## Conceptual framework

**Fig 1:**
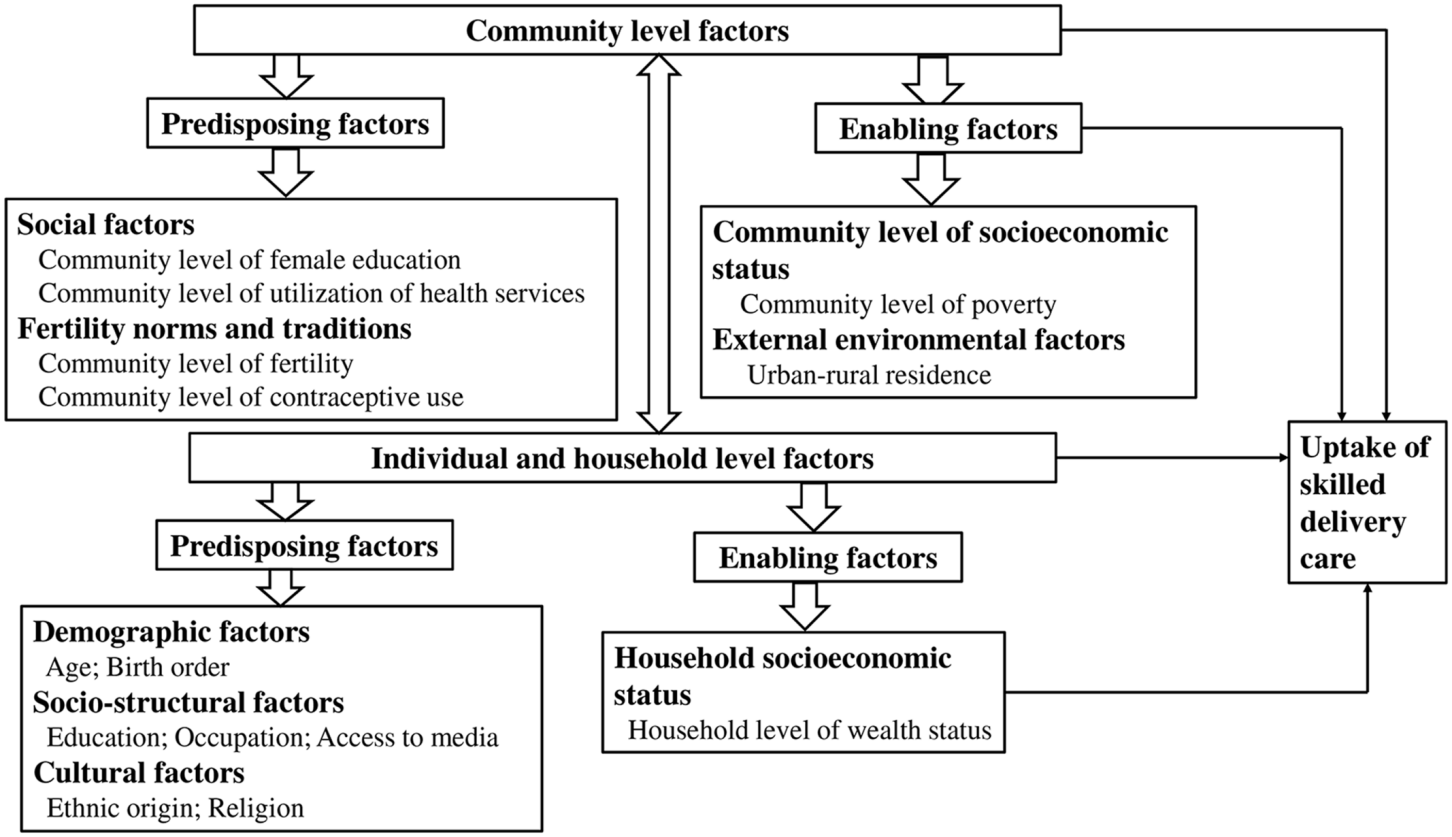
Conceptual framework of the study.

The conceptual framework of the study borrows constructs and variables from Anderson’s behavioral model of health service use [20]. Key strength of the model for the study lies on specifying need vis-à-vis predisposing and enabling factors at multiple levels that allows hierarchical analysis of macro and micro level determinants the study intends to explore in the context of uptake of skilled delivery care in Ethiopia. In this model, utilization of health services is an individual decision constrained or promoted by social structure and availability of health services. The framework is useful to modeling effects of hierarchical factors on individual outcome accounting for proximate as well as remote determinants at different levels. According to the model, health service utilization is a function of institutional factors (health care system measured in terms of health policy, organization and resources); external environmental factors comprising of social, economic and physical components; population factors that predispose individuals to use health services that include demographic, socio structural as well as health belief factors; enabling conditions of family and community resources; clinically evaluated or perceived need; health behavior that includes personal health practices and use of health care services, as well as health outcomes and consumer satisfaction [20]. The selection of variables for the framework adopted to suit the Ethiopian context in the current study was done reviewing existing empirical literature. The framework is given in Fig 1.

Justified in the hierarchical structure of factors presented in the model is the nested nature of individuals and households within communities. Contextual level predisposing factors presented in the model include community level of female education, community level of modern contraceptive use and community level of fertility although these conditions are not directly responsible for the uptake of maternal health services. Enabling factors include community level of poverty and external environmental factors such as urban- rural residence that facilitate or impede health service utilization.

These factors were measured aggregating individual level factors in order to identify possible effects of externalities. For example, mothers with a lower level of education may be positively influenced in their communities by other women with higher level of education which may lead to improved understanding of relevant information about safe motherhood and healthy practices in seeking skilled care during pregnancy and delivery. Likewise, poor women living in less poverty communities can also be thought to live in an empowered social and physical environment than those living in poverty stricken communities. And, this might encourage such women to seek care than their counterparts living in poorer communities. On the other hand, communities with high fertility may be more conservative in their attitudes and the expected roles of women which have implications in maternal health service utilization. Higher levels of uptake of family planning can also mean communities are less conservative towards traditional gender roles and norms that would allow women to have enhanced decision making power to seek care during pregnancy and labor.

The main groups of individual level predisposing factors identified as playing important part in the explanation of uptake of skilled delivery care are maternal age, birth order, education, occupation, cultural factors and media access. The individual level enabling factor identified as main determinants of the uptake of skilled delivery care used as a proxy for socioeconomic status, is household wealth. The unequal distribution of which constitute mechanisms by which differential in the uptake of such services are generated as a consequence of influences of factors operating at community and external environmental levels. The need factor considered for the study is a live birth during the five years preceding the survey.

## Materials and methods

### Data and sampling design

Data for the study is drawn from two rounds of Demographic and Health Surveys (DHS) from Ethiopia of the year 2005 and 2011. The surveys used two-stage stratified-cluster sampling design based on sampling frame of the 1994 and the 2007 Population and Housing Censuses respectively. Selection of clusters known as Primary Sampling Units (PSUs) was made in the first stage. The second stage involves selection of households from each cluster. Stratification in the first stage was achieved grouping the 11 federal regions into urban and rural areas. The 2005 survey had 540 (139 urban and 401 rural) PSUs selected using probability proportional to size sampling with 24–32 households per PSU. Furthermore, PSUs in regions with very small population were selected with equal size allocation. The year 2011 had 624 PSUs (187 in urban areas and 437 in rural areas) selected with a fixed number of 30 households in each PSU.

Sampling frame for the selection of households in the second stage was derived from household listing operation carried out in all the selected PSUs. The survey included a nationally representative sample of 14, 645 households and 14, 717 eligible women in the year 2005 and 18, 720 households and 17, 385 eligible women in the year 2011. The final sample size for this study consists of 6,544 women in the year 2005 and 7, 698 women in the year 2011 who had given a live birth in the five years preceding the surveys. For women who had more than one live birth, only the most recent live birth was considered. Details of sampling design and selection of sample are available in the Ethiopian Demographic and Health Survey reports from Measure DHS website (www.dhsprogram.com).

### Outcome variable

Outcome variable for the study is a binary response indicating whether a woman had given birth with the assistance of skilled health personnel (Doctor, Nurse or Midwife).

### Exposure variables

A number of exposure variables have been selected based on theoretical and empirical significance the description and measurement of which are given in Table 1.

**Table 1:** Description and measurement of exposure variables.

**Community level factors and their description**	
Community level of female education	Aggregate values of community level female education measured by the proportion of women with a minimum of primary level of education derived from data on respondent’s level of education categorized as: “<25% = Low”, “25%-50% = Moderate” and “50% = High education communities.”
Community level of poverty	Aggregate values of community level poverty measured by proportion of households in the poorest wealth quintile derived from data on wealth index categorized as: “<25% = Low”, 25%-50% = Moderate” and “>50% = High poverty communities.”
Community level of health service utilization	Aggregate values of community level of health service utilization measured by the proportion of women who had visited health facility in the past 12 months derived from the family planning module of DHS data categorized as: “<50% = Low” and “> 50% = High health service utilization communities.”
Community level of fertility	Aggregate values of community level of fertility derived from data on children ever born categorized as: “<2.5 = Low” and “>2.5 = High fertility communities” taking the mean value to fertility at national level.
Community level of modern contraceptive prevalence	Aggregate values of community level of modern contraceptive prevalence measured by proportion of women who are currently using modern contraceptive categorized as: “<50% = Low” and “>50% = High contraceptive prevalence communities.”
Type of place of residence	The variable place of residence recorded as rural and urban in the dataset was retained without change.
**Individual and household level factors and their description**	
Age Group	Re-coded in four categories with values of 0 for 15–19, 1 for 20–29, 2 for 30–39 and 3 for 40–49.
Birth Order	Re-coded with a value of 0 for first order birth, 1 for second order birth, 2 for third order birth, and 3 for birth order four and above.
Ethnic Origin	Re-coded in four groups with a value of 0 for Oromo, 1 for Amhara, 2 for Tigre and 3 for other ethnic Ethiopian categories as most of the groups in this category are small in number.
Religion	Re-coded in three categories with a value of 0 for Orthodox, 1 for Muslim and other religious groups (combining protestant, catholic, traditional and the other religious categories as most women in this category are small in number).
Maternal Education	Re-coded in three groups with a value of 0 for no education, 1 for primary education and 2 for secondary and above combining secondary and higher education categories together.
Maternal Occupation	Re-coded in five categories with a value of 0 for not working combining it with domestic household work category, 1 for sales and services combining it with professional/technical/managerial categories, 3 for skilled manual, 4 for unskilled manual and agricultural laborers combining it with agricultural self-employed categories.
Media Access	A composite variable created combining whether a respondent reads newspaper, magazine, listen to radio and watch TV with a value of 0 for no access if a women lacks access to all the three media; 1 for medium access if a woman has access to either of the three media; and 3 for high access to media if a woman has access to more than one media at least once a week.
Wealth Index	The datasets contained wealth index that was created using principal components analysis (PCA) coded 1 for “poorest”, 2 for “poorer”, 3 for “Middle”, 4 for “Richer” and 5 for “Richest.”

### Data analysis

Analysis was performed using IBM SPSS version 22. Bivariate analysis was performed to examine association between uptake of skilled delivery care and the selected background characteristics. The net effect of each independent variable after adjusting for potential confounders was examined using two-level multivariable multilevel logistic regression analysis. The multilevel model is more appropriate for hierarchically structured data such as the DHS to estimate the robust standard error [21]. We fitted model involving two levels (individuals nested in communities) as:

$$Log\left(\frac{\pi_{ij}}{1-\pi_{ij}}\right)=Y_{ij}=\beta_{0}+\beta_{1}X_{1ij}\ldots \ldots \ldots +\beta_{n}X_{nij}+e_{ij}\;\;\;\;e_{ij}\sim N\left(0,\sigma^{2}\right)$$

Y_ij_ = outcome variable for individual i in j group; X_ij_ = individual-level variable for the i^th^ individual in group j. e_ij_ = individual-level error assumed independent and normally distributed with mean zero and variance of σ^2^. This model evaluates relationships and variances at multiple levels [21]. The fixed effects were reported in terms of odds ratios (OR) with their P-values and 95% confidence interval (CI) after controlling potential confounders. Two-tailed Wald test (significance level of alpha equal to 5%) was used to determine statistical significance. The random effects were expressed in terms of community level variance (*δ*^2^). Intra class correlation coefficient (ICC) and proportional change in variance (PCV) were used to examine clustering and the extent to which contextual factors explain the unexplained variance of the empty model. The ICC was calculated as:

$$ICC=\frac{V_{A}}{V_{A}+3.29}.$$

Where: V_A_ is community level variance and 3.29 is individual level variance (V_I_) equal to π^2^/3 [22]. The PCV was calculated as:

$$PCV=\frac{V_{N-1}-V_{N-2}}{V_{N-1}}$$

Where: V_N-1_ is the neighborhood variance in the empty model and V_N-2_ is the neighborhood variance in the subsequent model [23].

### Ethical issues

Since secondary data from DHS provided by Marco International was used, there was no ethical approval required. It should however, be noted that Macro International obtained informed consent from individuals who participated in the DHS surveys.

## Results and discussion

### Factors associated with the uptake of skilled delivery assistance

#### Descriptive analysis

Among women who had a live birth in the five years preceding the surveys, 7% and 12% gave birth with the assistance of skilled health personnel in the year 2005 and 2011 respectively. Table 2 shows that in both the surveys the uptake of skilled delivery care decline by birth order. Skilled delivery care for first order birth was about 5 times higher than the four or higher order births. Utilization of skilled delivery care also varied according to the level of mother’s education. Uptake of delivery attended by skilled health personnel ranged from 3% in 2005 to 5% in 2011 among women with no education whereas among women with secondary or higher education it rose from 61% in 2005 to 74% in 2011. With respect to occupation, skilled birth attendance is lower for those mothers who were not working. Women who belong to richest wealth quintile receive higher percentage of skilled delivery care than their counterpart. Furthermore, the uptake of skilled delivery care has increased rapidly between 2005 and 2011 for women who belong to the richest wealth quintile and the increase was only marginal for women in the poorest wealth quintile. High access to media seems to have greater influence on the delivery care assisted by health personnel. For instance, in 2011, delivery assisted by skilled personal was about 54% to those who had high access to media compared to only 4% among those who did not have access.

**Table 2:** Patterns of uptake of skilled delivery care by women's characteristics: Bivariate analysis.

Individual and Household Characteristics	Year 2005		Total Women (n)	Year 2011		Total Women (n)
	Number	Percent		Number	Percent	
**Age group**						
15–19	28	7	430	37	9	394
20–29	312	9	3414	592	15	3933
30–39	118	5	2555	238	9	2720
40–49	45	5	867	45	6	784
Total	503	7	7266	912	12	7831
**Birth order**						
1st order birth	197	17	1162	344	26	1346
2nd order birth	126	12	1082	214	16	1335
3rd order birth	60	6	1000	146	13	1109
Birth order 4+	120	3	4021	208	5	4041
Total	503	7	7265	912	12	7831
**Education**						
No education	155	3	5717	246	5	5242
Primary	128	11	1188	404	18	2236
Secondary or higher	220	61	360	263	74	354
Total	503	7	7265	913	12	7832
**Occupation**						
Not working	300	6	5008	399	12	3484
Sales and Services	153	25	623	315	21	1464
Skilled manual	14	12	113	100	17	558
Unskilled manual and Agri workers	34	2	1509	87	4	2251
Total	501	7	7253	901	12	7757
**Ethnic Origin**						
Other Ethnic Ethiopians	137	6	2171	190	8	2303
Amhara	185	9	2116	385	17	2230
Oromo	141	6	2495	261	10	2739
Tigray	40	9	467	72	14	516
Total	503	7	7249	908	12	7788
**Religion**						
Orthodox	309	10	3233	556	17	3264
Muslim	94	4	2375	197	8	2563
Other religions	100	6	1658	158	8	1913
Total	503	7	7266	911	12	7740
**Wealth Status**						
Poorest	12	1	1514	35	2	1725
Poorer	23	2	1545	47	3	1688
Middle	30	2	1584	55	3	1605
Richer	74	5	1446	116	8	1490
Richest	365	31	1178	660	50	1325
Total	504	7	7267	913	12	7833
**Media Access**						
No access	117	3	4558	112	4	3150
Moderate access	293	11	2567	500	12	4112
High access	91	82	111	301	54	554
Total	501	7	7236	913	12	7816

Table 3 gives the uptake of skilled delivery care by community characteristics. As indicated in the table, delivery attended by skilled health personnel tends to be more common among women residing in urban areas in both the 2005 and 2011 surveys. Women residing in a highly-educated community received higher percentage (32% in 2005 and 27% in 2011) of skilled delivery assistance compared to community with poor female education (2% in 2005 and 1% in 2011). Women also reported higher uptake of skilled delivery care if they lived in communities where the proportion of households in the poorest wealth quintile is low. Similarly, the uptake of delivery care from skilled health personnel was greater where health service utilization is higher in both the survey years. Reported uptake of skilled delivery care was also higher among women who reside in the communities where fertility is low and contraceptive prevalence is high compared to communities where fertility is high and contraceptive prevalence is low in both the surveys.

**Table 3:** Patterns of uptake of skilled delivery care by community characteristics: Bivariate analysis.

Community Characteristics	Year 2005		Total	Year 2011		Total
	Number	Percent		Number	Percent	
**Type of Place of Residence**						
Urban	301	46	620	638	54	1187
Rural	202	3	6646	294	5	6716
Total	503	7	7266	932	12	7903
**Community Level of Female Education**						
<25%	58	2	3990	24	1	1674
25%–49%	109	5	2246	132	4	3393
>50%	336	32	1029	777	27	2837
Total	503	7	7265	933	12	7904
**Community Level of Poverty**						
>50%	7	1	859	15	1	1081
25.0–48.4%	9	1	1394	57	3	1702
<24.1%	484	10	4981	860	17	5106
Total	500	7	7234	932	12	7889
**Community Level of Health Service Utilization**						
Low utilization areas	140	3	4227	288	6	4782
High utilization areas	363	12	3039	644	21	3121
Total	503	7	7266	932	12	7903
**Community Level of Fertility**						
Low fertility areas	395	19	2107	759	27	2813
High fertility areas	108	2	5159	173	3	5090
Total	503	7	7266	932	12	7903
**Community Level of Modern Contraceptive Prevalence**						
Low contraceptive areas	112	3	4490	182	5	3976
High contraceptive areas	391	14	2776	750	19	3927
Total	503	7	7266	932	12	7903

#### Multivariable multilevel analysis

Table 4 shows results of the random intercept multilevel model. Model 1 (empty model) is with no covariates and model 2 is with covariates included. In the full model, the model accuracy was estimated at about 94% for 2005 and 93% for 2011. The results show that there is a considerable level of variation in the uptake of skilled assistance during delivery at the community level. As reported by intra-class correlation coefficient nearly 61% of the variation in the uptake of skilled delivery care is attributed to whether a woman resides in one community or another for both the 2005 and 2011 surveys. After adjusting for individual, household and community characteristics in the full model, the unexplained community level variation in the uptake of skilled delivery care were remained same with 22% and 21% respectively in the year 2005 and 2011. Despite the reduction of community level unexplained variance in model 2, there were still a significant level of unexplained variance presence at community level. This other unobserved influence could be such as supply side factors or other behavioral and cultural factors which were not included in this study due to unavailability of data at the community level.

**Table 4:** Community level clustering in uptake of skilled delivery care by survey year 2005 and 2011.

Model Term	Year 2005	Year 2011
Model 1		
Empty Model	(N = 6,538 Exc. = 6)	N = 7, 692 Exc. = 6)
Area variance	5.043(0.428)***	5.226(0.408)***
ICC	0.605	0.613
Model 2		
Model including all variables	(N = 6,455 Exc. = 89)	(N = 7,416 Exc. = 282)
Intercept	0.086(0.035–0.213)***	0.183(0.080–0.418)***
Variance Intercept	0.937(0.515)***	0.898(0.126)***
ICC	0.221	0.214
PCV (%)	0.634	0.651

**Individual and Household Level Factors:** Table 5 presents results of the multivariable multilevel logistic regression analysis by survey year 2005 and 2011. In the full model which control for individual, household and community level factors, there is a positive association between maternal age and the uptake of delivery assistance from skilled health personnel (doctor, nurse or midwife) in both the survey years. The results further indicate that the likelihood of skilled delivery assistance is higher among older mothers compared to younger mothers in both the survey years. For example, in the year 2011, women in the age group 20–29, 30–39 and 40–49 were nearly twice (OR = 1.972(95% CI = 1.220–3.188), (OR = 2.303 (95% CI = 1.352–3.922) and (OR = 2.299 (95% CI = 1.238–4.272) more likely to uptake the skilled delivery care than those in the age group 15–19. This is consistent with the results from other studies [7, 24, 25, 26, 27]. The finding that older women in Ethiopia had higher odds of skilled assistance during delivery could be attributed to the better awareness of availability and accessibility of such services. Older women also generally have a maternal health care experience than the younger women.

**Table 5:** Effects of individual and household level factors in uptake of skilled delivery care by survey year.

Model Term	2005 Model	2011 Model
	N = 6,455 Exc. = 89	(N = 7,416 Exc. = 282
	OR (95% CI)	OR (95% CI
**Intercept**	0.086(0.035–0.213)***	0.183(0.080–0.418)***
**Age group**		
15–19	1.000	1.000
20–29	1.293(0.820–2.307)	1.972(1.220–3.188)**
30–39	1.703(1.002–2.894)*	2.303(1.352–3.922)**
40–49	2.227(1.109–4.470)*	2.299(1.238–4.272)**
**Birth order**		
1^st^ order birth	1.000	1.000
2^nd^ order birth	0.431(0.311–0.596)***	0.573(0.427–0.769)***
3^rd^ order birth	0.360(0.243–0.533)***	0.417(0.303–0.572)***
Birth order 4+	0.223(0.150–0.332)***	0.339(0.244–0.471)***
**Educational level**		
No education	1.000	1.000
Primary education	1.582(1.200–2.086)**	1.797(1.431–2.257)***
Secondary or higher	2.937(2.018–4.275)***	4.423(3.093–6.325)***
**Maternal occupation**		
Not working	1.000	1.000
Sales & services	1.578(1.173–2.121)**	0.974(0.788–1.203)
Skilled manual	1.285(0.737–2.238)	1.060(0.722–1.556)
Unskilled manual & agri	0.752(0.497–1.136)	0.672(0.487–0.927)*
**Ethnic Origin**		
Other ethnic Ethiopians	1.000	1.000
Amhara	0.775(0.548–1.096)	1.076(0.792–1.461)
Oromo	0.713(0.495–1.026)	0.936(0.701–1.250)
Tigre	0.745(0.403–1.379)	0.585(0.372–0.918)*
**Religious categories**		
Orthodox	1.000	1.000
Muslim	0.902(0.657–1.240)	0.796(0.606–1.045)
Other religions	0.902(0.657–1.240)	0.708(0.491–1.021)
**Wealth quintile**		
Poorest	1.000	1.000
Poorer	0.796(0.395–1.605)	0.889(0.586–1.350)
Middle	0.994(0.517–1.913)	0.881(0.579–1.339)
Richer	2.109(1.135–3.919)*	1.281(0.858–1.914)
Richest	3.581(1.945–6.594)***	2.121(1.403–3.207)***
**Access to media**		
No access	1.000	1.000
Medium access	1.248(1.054–1.936)*	1.433(1.117–1.839)**
High access	2.832(1.648–4.867)***	2.237(1.590–3.146)***

Note: Community level factors have been controlled in both the models

A consistent negative association between the uptake of skilled delivery assistance and birth order is observed. The results indicate that women of birth order two, three, four and above had 42% (OR = 0.573(95% CI = 0.427–0.769), 58% (OR = 0.417(95% CI = 0.303–0.572) and 66% (OR = 0.339 (95% CI = 0.244–0.471) lower odds to have their birth attended by skilled health personnel in the year 2011. Similar gradient is observed in 2005. Similar finding was also observed in other studies [7, 10, 15, 25, 26, 28]. However, the finding that older aged women have higher odds of uptake of skilled delivery care services and on the other women of higher birth order have lower odds of uptake of such services was surprising since higher birth order women who would generally be older were less likely to seek these services than women of first order birth who would generally be younger. Similar results are, however, reported in studies conducted in Ethiopia and elsewhere [7, 15, 27].

The inverse association between birth order and the uptake of skilled assistance during delivery in Ethiopia could be related to quality of care and the importance given to lower order births. Even though the Ethiopian health system has witnessed substantial improvement in the recent decade, quality of the health service like in many other resource constrained setting is poor with critical shortages of health personnel, erratic supplies of drugs and equipment. Hence it can be argued that, in a traditional society like Ethiopia, one could easily lose confidence in the health system if a woman continues to suffer complications while attending maternity care. This could discourage women from seeking health services in later pregnancies and this could discourage the uptake of skilled delivery care as women join higher parity ranks.

The odds of uptake of skilled assistance during delivery significantly and positively increase with the educational attainment of a woman. Although the effect of education becomes stronger over the years, it is much stronger among women with secondary or higher level of education than those with primary level of education. For example, whereas women who have completed primary education are 1.5 times and 1.7 times more likely to have delivered attended by skilled health personnel in the year 2005 and 2011 respectively, those with secondary or higher level of education are nearly three times (OR = 2.937(95% CI = 2.018–4.275) and more than four times (OR = 4.423(95% CI = 3.093–6.325) respectively in 2005 and 2011 more likely to use skilled health personnel during delivery. Several other studies have reported similar results [5, 7, 11, 15, 28]. Moreover, this shows that the effect of education become stronger over the years.

Given the low level of women’s education and the inevitable lack of health knowledge coupled with the prevailing traditional beliefs in pregnancy and child birth in the country, the evidence that women with exposure to higher education had higher uptake of skilled assistance during delivery compared to those with no education is expected. It can be argued that in societies where health care is widely available women’s status may not affect use of health services. However, in countries where health care is a scarce commodity, women’s ability to use health services could increase with social status. The evidence that women working in sales and service sectors had higher odds of the uptake of skilled delivery assistance could give an indication that women’s earning ability is an important factor in the uptake of skilled delivery care in Ethiopia. Enhanced access to resources, wider social networks and growing understanding of their social environment could provide an impetus to seize opportunities available to them than women traditionally labeled housewives who are in the not working categories.

Another important factor that this study found was access to media which also had a consistent and positive effect on the uptake of skilled delivery assistance for both the survey years. For example, women living in households with moderate and high access to media have increased odds of receiving delivery assistance from skilled health personnel by 24% (OR = 1.248(95% CI = 1.054–1.936) and 183% (OR = 2.832(95% CI = 1.648–4.867) in the year 2005, the same was by 43% (OR = 1.433(95% CI = 1.117–1.839) and 123% (OR = 2.237(95% CI = 1.590–3.146) in the year 2011. Having access to information from modern media influences the women’s knowledge of health care needs during pregnancy and availability of such services [7, 15, 29]. This is especially true in a society marked by popular or non-scientific health beliefs, limited exposure to modern health services and the subsequent skeptical cultural attitude towards modern medical care and the relative high dependency on illness response in seeking care. Consequently, media can play a crucial role to bridging these knowledge gaps and promoting scientific attitudes thereby encouraging women to use health services not just when illness arises but for maintaining health.

The effect of wealth quintile seems to be weakening over the years. For example, women in the upper two wealth quintile (richer and richest households) were nearly twice (OR = 2.109(95% CI = 1.135–3.919) and more than three times (OR = 3.581(95% CI = 1.945–6.594) more likely to receive skilled delivery assistance in the year 2005, however, only women in the upper most wealth quintile (richest households) had more than twice (OR = 2.121(95% CI = 1.403–3.207) higher odds of uptake of maternal health services in the year 2011. Other studies also support this finding [28, 29, 30, 31]. This indicates that poverty continue to be an important determinant for non-use of skilled delivery assistance in Ethiopia, however, women from fifth quintile group (richest households) could afford the uptake of skilled delivery assistance compared to other quintile groups in the recent year.

In a resource constrained setting like Ethiopia, women might seek maternal health services; however, availability of financial resources is the actual means to obtaining such services. In one study, it was found that among facilities that provided delivery care, 68% charged fees for drugs and supplies in cash or kind and the average cost of normal and caesarean delivery was reported US$ 7.00 and US$ 51.80 respectively [32]. For women seeking delivery care, additional cost involves cost for transportation and opportunity cost in terms of travel time, waiting time and loss of productive activities [33]. Hence in such a context, household wealth, particularly among richest could enable women to translate potential access to realized access.

**Community Level Factors:** The results of the multilevel logistic regression analysis of community level factors controlling individual and household level factors by survey year are presented in the Table 6. Women’s community level female education has stronger effect on the uptake of delivery assistance by skilled personnel though the effect of this decrease over the years (see Table 6). The result was consistent with other studies elsewhere [9, 28, 34, 35]. The significant and positive effects of community level of female education on the uptake of skilled delivery assistance could suggest possible effects of externalities where the social environments in these communities positively influence the knowledge, attitude and practices of individual women in making their pregnancy safer through uptake of skilled care during delivery.

**Table 6:** Effects of community level factors on the uptake of skilled delivery care by survey year.

Model Term	2005 Model	2011 Model
	(N = 6,455 Exc. = 89)	(N = 7,416 Exc. = 282)
	OR (95% CI)	OR (95% CI)
**Intercept**	0.086(0.035–0.213)***	0.183(0.080–0.418)***
**Place of residence**		
Urban	1.000	1.000
Rural	0.340(0.196–0.591)***	0.169(0.116–0.247)***
**Area level of female education**		
24.5%	1.000	1.000
25.0–48.7%	1.898(1.181–3.049)**	1.223(0.790–1.892)
>50%	3.414(1.671–6.972)**	1.744(1.086–2.800)*
**Area level of poverty**		
>50%	1.000	1.000
25.0–48.4%	0.712(0.292–1.739)	0.973(0.584–1.622)
<24.1%	1.140(0.516–2.157)	1.059(0.644–1.740)
**Area level of utilization of health services**		
Low utilization areas	1.000	1.000
High utilization areas	1.302(0.951–1.783)	1.225(0.921–1.613)
**Area level of fertility**		
Low fertility areas	1.000	1.000
High fertility areas	0.568(0.372–0.867)**	0.605(0.435–0.842)**
**Area level of contraceptive use**		
Low contraceptive. use areas	1.000	1.000
High contraceptive. use areas	0.858(0.592–1.244)	1.328(0.967–1.825)

Note: Individual and household level factors have been controlled in both the models.

As expected, women residing in rural communities were less likely to use skilled delivery assistance by 66% (OR = 0.340(95% CI = 0.196–0.591) in 2005 and 83% (OR = 0.169(95% CI = 0.116–0.247) in 2011. The results also show that the disparity between rural and urban in the uptake of delivery assisted by health personnel is increasing over the years. The result is consistent with studies done in Ethiopia and elsewhere [5, 24, 26]. Several reasons could explain why the uptake of skilled delivery care is higher among urban compared to rural women in Ethiopia. The scattered nature of settlement patterns, the relative level of underdevelopment and limited supply of health facilities in rural communities could hamper the uptake of such services in these communities. On the other hand, the shorter distance to health facilities in urban areas given the good road infrastructure and transportation facilities and the relative ease of access to information and media in these areas could also facilitate better diffusion of modern medical services than in rural areas.

The results from this study also showed living in high fertility communities significantly reduce the odds of uptake of delivery assistance from skilled health personnel. Other studies have shown similar results elsewhere in the similar context [10, 30, 35]. It can be argued that communities with high fertility may be more conservative in their attitudes toward the expected roles of women which have implications on maternal health service utilization. Furthermore, higher child-dependency ratio per family may lead to structural barriers related to cost and time which may prevent mothers from seeking health services [36].

## Conclusions and policy implications

The purpose of this paper was to understand the individual, household and contextual determinants of skilled delivery assistance in a poor resource setting such as Ethiopia. The evidence showed that community level of female education and community level of fertility significantly predict the uptake of skilled delivery care. This reinforces the broad need for community empowerment. Thus, ensuring the provision of social services focusing on less privileged communities should eventually increase the uptake of skilled delivery care. The evidence that community level of fertility is a determinant factor in uptake of skilled delivery assistance also warrants the need to ensure universal access to family planning services focussing high fertility communities.

Equally important individual level factors affecting the uptake of skilled delivery assistance should be considered for evidence based programming. At individual level, the findings suggest efforts to promote the uptake of skilled delivery care should pay special attention to the needs of the youth, multiparous, less-educated and women in the poorest households. For example, the evidence that women discontinue the uptake of skilled assistance during delivery as they join higher parity is an indication of the existence of missed opportunities that reinforce the need for targeted promotion of these services among these demographics as major priority interventions.

The evidence that woman’s education and household wealth are key factors influencing the uptake of skilled assistance during delivery also reinforces any strategy targeted to address maternal health in the country need to take into account the prevailing disparities by women’s education and wealth. Thus, while policies and programs to foster women’s empowerment and their increased upward social mobility remain central to promoting maternal health in a mid to a long term, community based intervention strategies to educating and counseling mothers on safe motherhood could be of paramount importance in a short run. Similarly, the evidence that socioeconomic status is the most important factor associated with the uptake of skilled delivery care in Ethiopia warrant use of one or more techniques tried in some settings that are effective to empowering poor mothers and creating effective demand for such services in the country. Among others, such interventions could be conditional cash transfers, voucher programs and social marketing.

The study also noted that the effect of individual, household and community factors on the uptake skilled delivery care is changing over time in Ethiopia. It was found that the differences widened between rural and urban areas. Also, individual level women’s education becoming a stronger predictor whereas the effect of community level women’s education weakening over time. At the household level, the wealth gradient in the uptake of skilled delivery care is disappearing over the years except in the richest households.

To conclude, the study results have documented factors affecting the uptake of skilled delivery care in Ethiopia operate at multiple levels. This suggests the need to go beyond addressing challenges at individual and household levels to improve the uptake of such services. Pertinent to enhance maternal health seeking behaviour during delivery in Ethiopia is the need to consider community contexts in the design of maternal health programs. In this regard, while it is critical for policies and programs to improve and expand services, there is also a need to employ multi-sectoral approach to focusing on communities’ needs and realities as drivers of policies and programs to ensuring health services are available to those who are in greater need of them. For example, given that skilled delivery care is offered at secondary or tertiary levels of the health system which are further away from communities in the Ethiopian health system, upgrading health posts to provide obstetric services could be important in bringing skilled delivery care closer to communities. Furthermore, the challenging terrains and the poor transport infrastructure in rural communities meant focus should be on expanding road links between rural communities and health facilities.

### Limitations of this study

Notwithstanding the strengths of the study, certain limitations are worth mentioning. First, the cross-sectional nature of the data does not allow drawing causal inferences. DHS data are also associated with recall bias given that data was collected retrospectively on events that took place five years before the survey. In order to mitigate this effect, analysis was based on the most recent birth in the five years preceding the survey.
